# Improving the Cryotolerance of Wine Yeast by Interspecific Hybridization in the Genus *Saccharomyces*

**DOI:** 10.3389/fmicb.2018.03232

**Published:** 2019-01-08

**Authors:** Estéfani García-Ríos, Alba Guillén, Roberto de la Cerda, Laura Pérez-Través, Amparo Querol, José M. Guillamón

**Affiliations:** Departamento de Biotecnología de los Alimentos, Instituto de Agroquímica y Tecnología de los Alimentos – Consejo Superior de Investigaciones Científicas, Valencia, Spain

**Keywords:** *Saccharomyces cerevisiae*, must fermentation, low temperature, winemaking, hybrids, *Saccharomyces uvarum*

## Abstract

Fermentations carried out at low temperatures (10–15°C) enhance the production and retention of flavor volatiles, but also increase the chances of slowing or arresting the process. Notwithstanding, as *Saccharomyces cerevisiae* is the main species responsible for alcoholic fermentation, other species of the genus *Saccharomyces*, such as cryophilic species *Saccharomyces eubayanus*, *Saccharomyces kudriavzevii* and *Saccharomyces uvarum*, are better adapted to low-temperature fermentations during winemaking. In this work, a *Saccharomyces cerevisiae* × *S. uvarum* hybrid was constructed to improve the enological features of a wine *S. cerevisiae* strain at low temperature. Fermentations of white grape musts were performed, and the phenotypic differences between parental and hybrid strains under different temperature conditions were examined. This work demonstrates that hybridization constitutes an effective approach to obtain yeast strains with desirable physiological features, like low-temperature fermentation capacity, which genetically depend on the expression of numerous genes (polygenic character). As this interspecific hybridization approach is not considered a GMO, the genetically improved strains can be quickly transferred to the wine industry.

## Introduction

In winemaking, fermentation at lower temperatures correlates with a fresh character and fruity notes in wines (Beltran et al., 2002; Torija et al., 2003; Molina et al., 2007). This is consequence of a higher preservation (less evaporation) of varietal and fermentative aroma (Mouret et al., 2014). The use of low temperature during the fermentation process improves product quality, but also prolongs the time needed to complete fermentation and, therefore, increases the economic cost and energy requirements. Low temperature is one of the most important environmental stresses that influences the life and distribution of living organisms. In the yeast *Saccharomyces cerevisiae*, reductions in environmental temperature have widespread effects on growth and survival and may play an important role in the imposition of *S. cerevisiae* versus non-*Saccharomyces* species during wine fermentation (Salvadó et al., 2011a,b). Generally, the non-*Saccharomyces* yeasts, predominant in grape juice, are rapidly outcompeted by *S. cerevisiae* because of their poor adaptation to increasing concentrations of ethanol, impoverishing of nutrients and the lack of oxygen (Torija et al., 2001; Beltran et al., 2002). However, this population dynamic can be modified by dropping the fermentation temperature, favoring the growth and survival of non-*Saccharomyces* species for a longer period (Salvadó et al., 2011b).

In any case, industry is clearly interested in developing yeast strains with an enhanced capability to ferment at low temperatures. Naturally cold-tolerant strains of the *Saccharomyces* genus, like *S. kudriavzevii*, *S. uvarum* or *S. eubayanus* could potentially be used for low-temperature fermentations but tend to have higher ethanol sensitivity than *S. cerevisiae* and may, therefore, be less suitable for alcoholic fermentation. Among these *Saccharomyces* cryotolerant species, only *S. uvarum* has been isolated from wine and cider fermentations, mainly related with low temperature processes (Naumov et al., 2000, 2001; Demuyter et al., 2004; Rodríguez et al., 2014, 2017). However, all these cryotolerant species contribute to winemaking through its presence in natural yeast hybrids. *S. uvarum* ×*S. cerevisiae* hybrid strains have been isolated from Italian wines (Masneuf et al., 1998), Hungarian Tokaj wines (Antunovics et al., 2005), and Alsacian wines in France (Demuyter et al., 2004; Le Jeune et al., 2007). A new type of hybrids that result from the hybridization between *S. cerevisiae* and *S. kudriavzevii* have also been described among wine strains (González et al., 2006; Lopandic, 2009; Erny et al., 2012; Peris et al., 2012). Hybridization process between *Saccharomyces* species has been proposed as an adaptation mechanism to different stresses but especially to low temperature (Sipiczki, 2008). The hybrids described on wine have the physiological capability from both parental. Hybrids might have inherited the ability to grow at high temperatures (30–37°C) and ethanol tolerance from their *S. cerevisiae* parentals and ability to grow at low temperatures (10–16°C) from their *S. kudriavzevii*, *S. uvarum*, and *S. eubayanus* parentals (Belloch et al., 2008; Gamero et al., 2013; Alonso del Real et al., 2017; Magalhães et al., 2017a,b; Origone et al., 2018). Thus, a possible biotechnological solution for improving cryotolorance in wine yeasts is the generation of artificial interspecific hybrids into the *Saccharomyces* genus. Although, it can be found previous reports about the generation of artificial hybrids between *S. cerevisiae* and *S. uvarum* (Kishimoto, 1994; Rainieri et al., 1999; Sebastianini et al., 2002; Solieri et al., 2005; Origone et al., 2018), we decided to exploit this tool by using a different methodological approach for the hybridization and stabilization of the hybrids. In our study, the best parental for providing the cryotolerance character to the new hybrid was based on a comprehensive phenotypic evaluation of the temperature tolerance of a large collection of isolates, belonging to different species, and running a competition experiment with the fittest strains. The estimation of the temperature range in which microorganisms grow better was not only important for selecting the parental strain of the hybrid yetalso for getting insight about the use of some of these species in the fermentation processes. The enological features of the new hybrid, mainly its fermentation capacity at low temperature, were evaluated in both synthetic (SM) and natural (NM) grape musts.

## Materials and Methods

### Yeast Strains and Media

In this study, 59 yeast strains belonging to genera *Saccharomyces* [(*S. eubayanus*, *S. kudriavzevii* and *S. uvarum*, previously describes as cryotolerant), *S. cerevisiae* as a good fermentative species] and *Kluyveromyces*, *Torulaspora*, and *Metschnikowia* (Sipiczki et al., 2018) as controls of other species used in fermentation, were used. All the strains employed in the study are detailed in Supplementary Table S1. The industrial strains were kindly provided by Lallemand Inc. (France). These strains were codified as ADY and were named from ADY1 to ADY59. Inocula were prepared by introducing one single colony from the pure cultures of each strain into 5 mL of the same medium to be used in the experiments (YPD, LM, SM or NM). After a 24-h incubation of the precultures at 28°C, the volume required to obtain a concentration of about 10^6^ cells mL^-1^ was inoculated in different media, as described below. The correct inoculation size was always confirmed by the surface spread on YPD agar plates.

The growth media selected for the experiments were YPD (glucose 20 g L^-1^, peptone 20 g L^-1^, yeast extract 10 g L^-1^), a mineral media derived from that described by Verduyn et al. (1990), hereafter referred to as the lab medium (LM), and synthetic grape must (SM). The latter was derived from synthetic grape must (pH 3.3), as described by Riou et al. (1997), but with 200 g L^-1^ of reducing sugars (100 g L^-1^glucose and 100 g L^-1^ fructose). The following were utilized: organic acids, malic acid 5 g L^-1^, citric acid 0.5 g L^-1^ and tartaric acid 3 g L^-1^; mineral salts KH_2_PO_4_ 750 mg L^-1^, K_2_SO_4_ 500 mg L^-1^, MgSO_4_ 250 mg L^-1^, CaCl_2_ 155 mg L^-1^, NaCl 200 mg L^-1^, MnSO_4_ 4 mg L^-1^, ZnSO_4_ 4 mg L^-1^, CuSO_4_ 1 mg L^-1^, KI 1 mg L^-1^, CoCl_2_ 0.4 mg L^-1^, H_3_BO_3_ 1 mg L^-1^ and (NH_4_)_6_Mo_7_O_24_ 1 mg L^-1^; vitamins myoinositol 20 mg L^-1^, calcium pantothenate 1.5 mg L^-1^, nicotinic acid 2 mg L^-1^, chlorohydrate thiamine 0.25 mg/L, chlorohydrate pyridoxine 0.25 mg L^-1^ and biotin 0.003 mg L^-1^. The assimilable nitrogen source used was 300 mg L^-1^ (120 mg L^-1^ as ammonium and 180 mg L^-1^ in the amino acid form). The sporulation medium was KAc (potassium acetate 1%, agar 2%).

### Growth Conditions

Growth was monitored by determining optical density at 600 nm in a SPECTROstar Omega instrument (BMG Labtech, Offenburg, Germany). Measurements were taken every 30 min for 4 days after 20-s of pre-shaking for the 25–40°C experiments, and every 90 min for 7 days for the low-temperature assays. Microplate wells were filled with the required volume of inoculum and 0.25 mL of the YPD or SM medium to always ensure an initial OD of approximately 0.1 (inoculum level of about 10^6^ cells mL^-1^). For each experimental series, the non-inoculated wells were also included in the microplate to determine, and to therefore subtract, the noise signal (García-Ríos et al., 2014). All the experiments were carried out in triplicate. Growth parameters were calculated from each treatment by directly fitting OD measurements *vs.* time to the reparametrized Gompertz equation proposed by Zwietering et al. (1990):

$$y\;=\;D^{*}\exp\{-exp[{\left(({\mu_{\text{max}}}^{*}\text{e})/D\right)}^{*}(\lambda \;-\;\text{t})+1]\}$$

where *y* = ln(OD_t_/OD_0_), OD_0_ is the initial OD and OD_t_ is the OD at time *t*; D = ln(OD_t_/OD_0_) is the asymptotic maximum, μ_max_ is the maximum specific growth rate (h^-1^) and λ is the lag phase period (h) (Aguilera et al., 2007). Overall yeast growth was estimated as the area under the OD vs. time curve (70 h and 168 h at 28°C and 15°C, respectively). This parameter was calculated by integration using the OriginPro 8.0 software (OriginLab Corp., Northampton, MA, United States).

### Competition Experiments

The best 10 strains, selected for their μ_max_ at 15°C, were inoculated together in a flask to reach an OD_600__nm_ of approximately 0.2 in total (0.02 OD_600nm_ per strain) in order to mimic the population size used in the wineries and to follow a complete growth curve. Cultures were allowed to grow through a normal growth curve, with a transfer of a small volume (the volume required to once again inoculate at an OD_600nm_ of 0.2) of the expanded culture in 60 mL of fresh medium every 3 days. Culture growth was monitored by measuring absorbance at 600 nm every 24 h. After each transfer, cultures were plated on solid YPD and 50 colonies of each point were randomly selected and genotyped by a mitochondrial DNA restriction analysis (Querol et al., 1992).

### Generation of Natural Auxotrophic Colonies From Parental Yeasts

The strains with the highest cell percentage in the competition experiments were selected to construct hybrids with other strains. All the selected strains were grown on 15 mL of YPD for 5 days at 28°C. Aliquots of each culture were seeded onto α-aminoadipic (α-AA) and 5-fluoroanthranilic acid (5-FAA) agar plates to select the lys- and trp- natural mutant colonies, respectively (Zarett and Sherman, 1985; Toyn et al., 2000). In order to confirm the presence of auxotrophy, the colonies that were able to grow were once again plated on new plates of MM [Yeast Nitrogen Base (YNB, Difco)], supplemented with 20 g L^-1^ of glucose as the carbon source and with 5 g L^-1^ of ammonium sulfate as the nitrogen source), α-AA and 5-FAA for 48–72 h at 28°C following the methodology proposed by Zarett and Sherman (1985), and partially modified by Pérez-Través et al. (2012).

### Rare-Mating

Rare-mating assays were carried out according to the procedures proposed by Spencer and Spencer (1996), with some modifications (Pérez-Través et al., 2012). The strains carrying the auxotrophic markers were grown separately in 25 mL GPY broth for 48 h at 28°C. Cells were recovered by centrifugation (4,000 × *g* for 5 min at room temperature), and the pairs of yeast cultures to be hybridized were placed together in the same tube. Aliquots of these mixed strains were inoculated in 2 mL of fresh YPD medium. After 5–10 days of static incubation in the slanted position at 28°C, cells were recovered by centrifugation (4,000 ×*g* for 5 min at room temperature), washed in sterile water, re-suspended in 1 mL of PBS and incubated for 2 h. A heavy suspension of the mixed culture was spread on the MM plates and incubated at 28°C. Prototrophic colonies usually appeared after 3–5 days. These colonies were isolated and purified by restreaking on the same medium (MM). The hybrid nature was confirmed by the PCR amplification of the *MAG2* and *GSY1* protein-encoding nuclear genes, and the subsequent RFLP analysis with restriction enzymes *Msp*I and *Taq*I (FastDigest, Thermo Scientific) (González et al., 2007).

### Genetic Stabilization and Ploidy Estimations of Hybrid Segregants by Flow Cytometry

The stabilization process was done by yeast sporulation. This process was induced by incubation on acetate medium for 5–7 days at 28°C. Following the preliminary digestion of the asci walls with 2 mg mL^-1^ glucuronidase (Sigma), viability was calculated as the percentage of spores (from a total of 40 analyzed spores per hybrid strain) able to form a colony on YPD agar after 48–72 h at 28°C. The hybrid nature was confirmed by the PCR amplification of the *MAG2* and *GSY1* protein-encoding nuclear genes, and the subsequent RFLP analysis with restriction enzymes *Msp*I and *Taq*I (FastDigest, Thermo Scientific) (González et al., 2007). Each selected spore was individually growed into 5 mL of YPD and incubated at 25°C. After reaching the stationary phase, an aliquot was used to inoculate a new tube. After five successive complete growths, an aliquot was plated on YPD plates and incubated at 25°C. Ten yeast colonies were randomly picked and characterized by inter-δ sequences (Legras and Karst, 2003) and RAPD-R3 (Pérez-Través et al., 2012) analyses. Simultaneously, the same colonies were inoculated in YPD and 10 colonies from each new culture were analyzed by the same methods. We considered a genetically stable spore when the colonies recovered after individual growths maintained the same molecular pattern than the previously inoculated (original) culture (Pérez-Través et al., 2012, 2014).

The DNA content of each parental, hybrid (H1) and segregant strains (S1-S3) was assessed by flow cytometry in a Beckman Coulter FC 500 (Beckman Coulter Inc., Brea, CA, United States) by the SYTOX Green dye method described in Haase and Reed (2002). The DNA content values were scored based on the fluorescence intensity compared with the *S. cerevisiae* diploid (FY1679) reference strain. The DNA content value reported for each strain was the result of two independent measures (Table 1).

**Table 1:** DNA content of the hybrids and parental strains.

Strains	DNA content
FY1679	2 0.08
ADY18	2.15 0.15
ADY59	2.22 0.01
H1	4.09 0.08
S1	3.23 0.03a
S2	3.11 0.04a
S3	3.25 0.01a


*Values expressed as mean ± standard deviation. ^a^ Indicates significantly different values (ANOVA and Tukey HSD test, α = 0.05, n = 2) compared with the parental strains.*

### Fermentations Trials

Fermentations were performed at 28°C and 15°C with continuous orbital shaking at 100 rpm. Fermentations were done in laboratory-scale fermenters using 100 mL bottles filled with 80 mL of SM or natural grape-must (NM). Dimethyl dicarbonate (DMDC) at 1 mL L^-1^ was added for sterilization purposes in NM. The NM used was Merseguera must, whose content was 173.45 g L^-1^ fermentable sugar, and nitrogen levels were 182 mg L^-1^. Flasks were closed with stoppers and airlocks to release CO_2_. Fermentation was monitored by mass loss until a constant mass was reached, considered to be the end of fermentation. Experiments were carried out in duplicate. Yeast cells were removed by centrifugation and supernatants were stored at -20°C until further analyses. Potential contaminations were monitored with a negative control (NM without cells). The monitored mass loss was corrected to the percent of sugar consumed, as in Pérez-Través et al. (2015):

$$C\;=\;\left(m^{*}[S\;-\;R]/(mf^{*}S)\right)*100$$

where *C* is the percent of sugar consumed at each time point, *m* is the mass loss value at that sampling time (g), *S* is the initial sugar concentration in the must (g L^-1^), *R* is the final sugar concentration in the fermented must (residual sugar, g L^-1^) and *mf* is the total mass loss value at the end of fermentation (g). The residual sugar values were obtained by the HPLC analysis of the fermentation samples. Curve fitting was carried out using the reparametrized Gompertz equation proposed by Zwietering et al. (1990).

### HPLC Analysis

Extracellular glucose, fructose, glycerol and ethanol were analyzed at the end of the SM and NM fermentations. Analytical HPLC was carried out in a Surveyor Plus Chromatograph (Thermo Fisher Scientific, Waltham, MA, United States) equipped with a refraction index detector, an autosampler and a UV–Visible detector. Prior to injection, samples were centrifuged at 13,000 rpm for 5 min, and then diluted 10-fold and filtered through 0.22 μm pore size nylon filters (Micron Analitica, Spain). A total volume of 25 μL was injected into a HyperREZ XP carbohydrate H + 8 mm column (Thermo Fisher Scientific) assembled to its correspondent guard. The mobile phase was 1.5 mM H_2_SO_4_ with a flux of 0.6 mL min^-1^ and a column temperature of 50°C. The concentration of each compound was calculated using external standards. Each sample was analyzed in duplicate.

### Statistical Analysis

HPLC data were analyzed by the Statistica 7.0 software package (StatSoft, Tulsa, OK, United States) by a one-way ANOVA and a Tukey test for the means comparison. The cytometry results were tested by a one-way ANOVA and a Tukey HSD test (α = 0.05, *n* = 2). Phenotypic data were fitted to the reparametrized Gompertz model by non-linear least- squares fitting using the Gauss–Newton algorithm as implemented in the nls function of the RStudio statistical software, v.1.1.456. Dendrograms were created using the hclust package in the RStudio statistical software, v.1.1.456.

## Results

### Effect of Temperature on Yeast Growth

**FIGURE 1:**
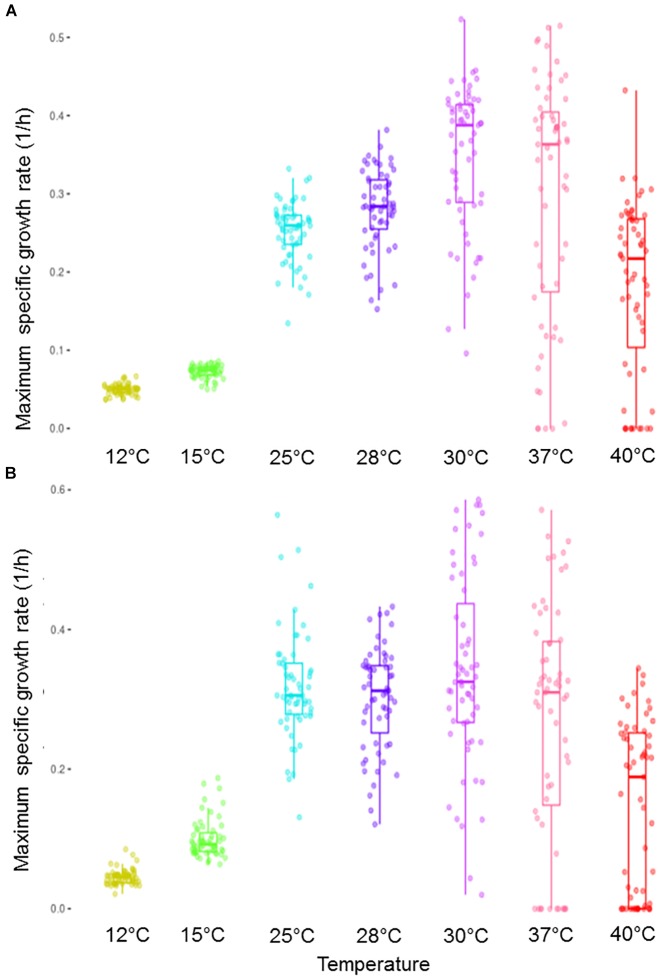
Box plot representation of the μ_max_ distribution in all the strains within the assayed complete temperature range. Growth was performed in a synthetic must **(A)** and in a complete lab medium **(B)**.

A phenotypic analysis was performed at different temperatures (12–40°C) to evaluate the thermotolerance differences of a collection of yeast strains belonging to diverse environmental niches and species. For this purpose, two different media, synthetic must (SM) and a complete laboratory medium (LM), were used. Figure 1 shows the maximum specific growth rate (μ_max_) of the complete set of yeasts for the whole range of assayed temperatures. For these temperatures, the average of the optimum growth temperature of all these strains could be fixed at around 33°C for both media. In addition, the higher the temperature was, the wider variance became.

**FIGURE 2:**
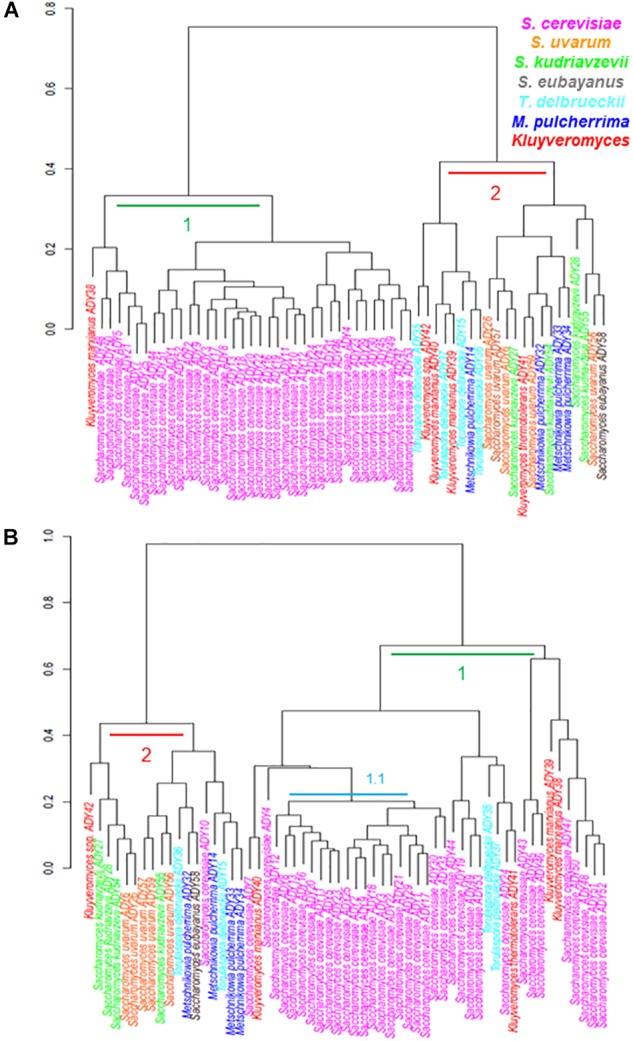
Dendrograms showing the phenotypic relationships among different strains according to temperature behavior in the synthetic must **(A)** and the complete lab medium **(B)**. Data was provided in form of distance matrix.

Figure 2 represents the phenotypic clustering of the complete set of strains assayed and grouped in growth behavior terms at all the tested temperatures. In both media, we observed two major groups (1 and 2) with some differences in their strain compositions. In SM (A), group 1 was integrated by all the studied *S. cerevisiae* strains and one strain belonging to *K. marxianus*. Group 2 was a mixture formed with the other study strains. Regarding LM (B), a dendrogram divided the strains once again into two groups (1 and 2) but, in this case, the separation between *cerevisiae*, non-*cerevisiae* and non-*Saccharomyces* was not as obvious as in SM. Group 1 was integrated mainly by all the *S. cerevisiae* strains, but some strains of genera *Torulaspora* and *Kluyveromyces* clustered together with them in this group. Thus the most homogenous group was subcluster 1.1, integrated by all the wine strains of *S. cerevisiae*. This phenotypic grouping is clearly determined by a combined effect of the growth medium and temperature. However, to the best of our understanding, the medium effect is stronger in SM, which separated *S. cerevisiae*, the most competitive species in a high-sugar medium such as SM, of the remainder strains, belonging to less competent species in this medium. In fact, the top 10 strains with higher μ_max_ at 15°C in SM belonged to *S. cerevisiae* (except *Kluyveromyces* spp. ADY42; Supplementary Table S2). Conversely, LM seems to be a less stressful medium, in which growth is mainly determined by the fitness at different temperatures. In LM, Cluster 1 was mainly integrated by the strains able to grow at 40°C (Supplementary Table S2), that is, the most tolerant strains at high temperature.

**FIGURE 3:**
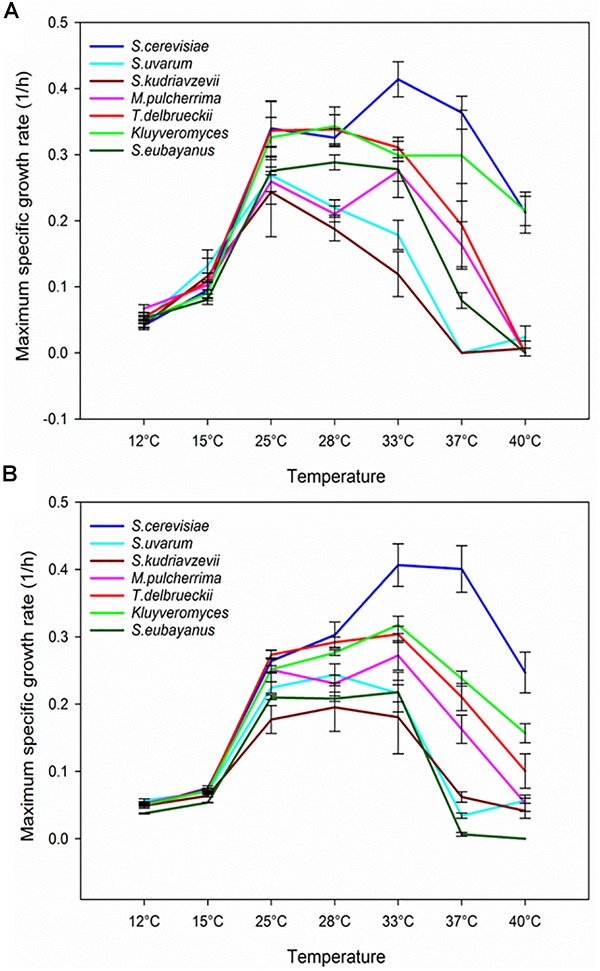
Maximum specific growth rate of each tested species within the whole assayed range of temperatures in SM **(A)** and the complete lab medium **(B)**. Values are expressed as the mean of the μ_max_ of all the strains belonging to the same species.

When the μ_max_ was calculated by species (Figure 3), practically all the strains showed a similar growth rate at 12–15°C and with very narrow differences up to 25°C. However, the temperature increases above 25°C provoked the greatest differences between *S. cerevisiae* and the remainder species, mainly in SM. This result is very interesting because demonstrates that the high temperature could be a more determining factor in the well-known capacity to outcompete to other species than the ethanol tolerance, as Salvadó et al. (2011a) already concluded. Recently, some species of non-*Saccharomyces* have drawn the attention of winemakers, as they positively modify the wine chemical composition, and consequently, improve their flavor and bouquet. Nowadays, different strains of *K. marxianus*, *M. aff. pulcherrima* and *T. delbrueckii* are available in the market to be co-inoculated or sequentially inoculated with *S. cerevisiae* strains. Our data evidenced that low temperature fermentations could be a good approach to increase their survival and contribution to the final wines.

### Competition Experiment

**FIGURE 4:**
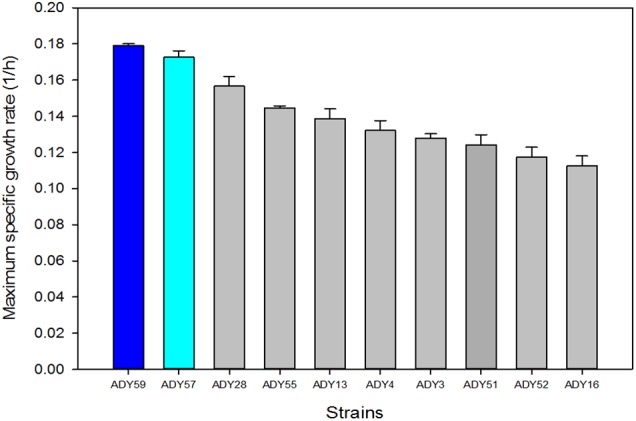
Maximum specific growth rate (h^-1^) of the best 10 *Saccharomyces* strains grown at 15°C in the complete lab medium (LM). Dark and light blue highlight the strains with the highest cell percentages in the competition test.

In order to validate the competitiveness of the best-adapted strains at low temperature, we selected the top 10 strains showing higher μ_max_ values of the phenotyping done at 15°C in LM (Supplementary Table S2). As mentioned above, we selected LM to avoid any interference from other stresses, such as low pH, high sugar content and high ethanol, which happened with the SM medium. The best 10 strains belonged to species *S. uvarum*, *S. kudriavzevii*, *S. cerevisiae*, and *T. delbrueckii*. This latter species was represented by the strain *T. delbrueckii* ADY35 that showed the best μ_max_ of all the tested strains at 15°C. However, as our aim was to select the best parental for crossing with *S. cerevisiae* strains, we only selected the best 10 strains belonging to *Saccharomyces* genus for the competition experiment (Figure 4). The strain dynamic in the subsequent batch-cultures was analyzed by the restriction of the mitochondrial DNA of the isolated colonies in the different competition experiment stages. The result of this strain imposition was the presence of two *S. uvarum* strains (ADY57 and ADY59; Supplementary Table S1) throughout the process, which co-existed in the culture, with percentages of 45 ± 2.5 and 55 ± 1.4%, respectively. ADY57 was isolated from Tokaji wine in Hungary, while ADY59 (Rodríguez et al., 2014; Origone et al., 2018) was isolated from apple Chicha fermentation in Argentina. It is noteworthy that the two *Saccharomyces* strains that outcompeted the others were those with the highest μ_max_ values at 15°C (Figure 4). The competition experiment was also run at 28°C as the control condition and the yeast that reached 100% of cells in culture was the commercial *S. cerevisiae* strain ADY16.

### Hybrids Generation and Characterization

We selected the spontaneous auxotrophic mutants of *S. cerevisiae* ADY18 (trp^-^) and *S. uvarum* ADY59 (lys^-^) as parental strains to perform hybridization to obtain a new hybrid strain with improved fermentation capacity at low temperature. ADY18 is a *S. cerevisiae* strain with very good enological features, but with the potential for improving its cryotolerance. *S. uvarum* ADY59 was the best strain in terms of μ_max_ and competitiveness at 15°C. After the rare-mating assay, the putative hybrids (prototrophic colonies) were checked by PCR and the positive ones were sporulated for a quick stabilization of its genome. After sporulation, the hybrid nature of the viable spores was again confirmed by the PCR amplification of the *MAG2* and *GSY1* protein-encoding nuclear genes, and the subsequent RFLP analysis with restriction enzymes *Msp*I and *Taq*I. In this process, we obtained three viable spores (S1–S3) out of 40 (7.5% of viability). The stable status of the spores was tested by inter-δ sequences and RAPD-R3 after successive rounds of batch-cultures. DNA content analysis showed that S1–S3 were 3n (likely alloaneuploid) derived from a tetraploid hybrid (H1). This ploidy of the hybrid confirmed us that it was formed by a rare-mating event and not by a spore-spore cross. Moreover, we also checked that the parental strains were not able to sporulate under the rare-mating assay.

### Fermentation Performance in the Synthetic Must

**FIGURE 5:**
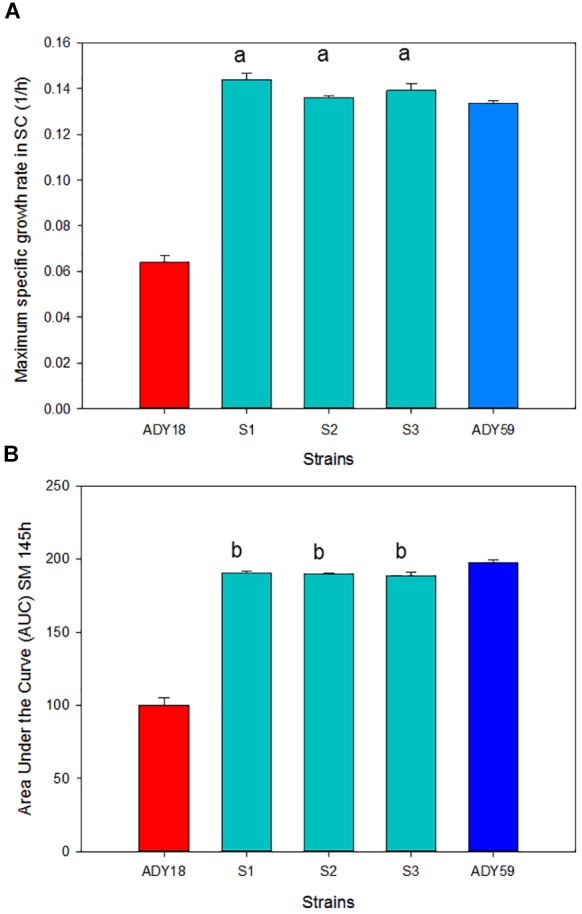
Growth parameters of the three segregants compared with parental strains *S. cerevisiae* ADY18 and *S. uvarum* ADY59 in the synthetic must (SM). **(A)** Maximum specific growth rate and **(B)** area under the curve (AUC). ^a^ Significant differences compared with the ADY18 strain. ^b^ Significant differences compared with both parental strains.

As a first selection step, all the stable spores (S1–S3), along with the two parental strains, were evaluated for growth parameters (Figure 5) and fermentative features (Table 2) in SM. Figure 5 shows the μ_max_ (Figure 5A) and the area under the curve (Figure 5B) of the three segregant spores compared with that of the parental strains at 15°C. The three segregants showed more than double the growth rate comparing with the *S. cerevisiae (Sc)* parental, and a slightly better value compared with the *S. uvarum (Su)* parental. The area under the curve (AUC) parameter allowed us to estimate overall behavior in all the growth phases. In relation to this parameter, hybrids obtained similar values compared with that of the *Su* parental, which almost doubled the *Sc* parental AUC value. As for the time needed to consume 50% and 100% of the sugars present in the must (Table 2), it should be noted that the three hybrid segregants showed lower T50 and T100 than both parentals at 15°C. The biggest differences between the *Sc* parental and segregants were observed in T50 because these spores have a much shorter lag phase than the industrial *Sc* strain (data not shown). At 28°C, segregants presented a similar fermentation kinetics compared with the *Sc* parental, while the *Su* parental presented a significantly delayed fermentation process as it was unable to consume the total amount of sugars and produced a stuck fermentation.

**Table 2:** Time (hours) required to consume 50% (T50) and 100% (T100) of the sugar content in SM and the Merseguera grape must (NM).

		ADY18	ADY59	S1	S2	S3
**Synthetic must**						
**15°C**	**T50**	219.96 0.00	193.91 5.61	162.46 6.78c	152.58 3.81c	167.40 4.12c
	**T100**	554.18 7.62	601.68 1.79	529.92 0.45c	525.88 5.72	531.67 4.83c
**28°C**	**T50**	33.74 1.33	41.43 0.00	31.82 0.67b	31.05 1.15b	30.67 0.67c
	**T100**	196.99 3.26	252.40 2.26	210.82 4.89b	212.55 4.07b	205.63 2.44b
**Merseguera**						
**15°C**	**T50**	126.64 3.26	97.45 3.52	115.12 0.00c	150.84 1.63c	131.26 0.01b
	**T100**	413.57 1.63	446.96 0.05	400.90 0.00c	403.20 6.52b	397.83 5.32c
**28°C**	**T50**	38.58 0.55	41.45 1.46	37.62 1.11b	51.66 0.00c	36.99 1.46b
	**T100**	198.09 0.96	199.04 0.96	188.87 2.21c	198.72 0.55	188.52 1.35c


*Values expressed as mean ± standard deviation. ^a^ Indicates significantly different values (p-value ≤ 0.05) compared with ADY18. ^b^ Indicates significantly different values (p-value ≤ 0.05) compared with ADY59. ^c^ Indicates significantly different values (p-value ≤ 0.05) compared with both parentals.*

### Fermentation Performance in the Merseguera Grape Must

To confirm the data obtained in SM, fermentations were also carried out in the natural grape must of the Merseguera white variety. As in SM, the kinetic growth curves of the NM fermentations were inferred by following mass loss. Growth curves were used to extract the kinetic parameters of each strain (Table 2). The Merseguera fermentations performed by segregants at 15°C were faster than both parentals. These differences were especially greater with the *Su* parental, which required around 47 h more to finish the process. According to its cryotolerance, this strain (*Su*) started fermentation very quickly, with the shortest lag phase and the smallest T50. However, it was more sensitive to the increasing ethanol concentration and showed a delay in the last fermentation stages. The segregant strains (S1–S3) also had a shorter lag phase than the *Sc* parental, but sustained good fermentation activity by the end of fermentation, perhaps as a consequence of improved ethanol resistance in comparison with the *Su* parental. At 28°C, S1 and S3 also showed a faster fermentative kinetics compared with both parentals. The differences among the three segregants were noteworthy, and we highlight the importance of each parental’s DNA distribution. The concentrations of glucose, fructose, glycerol and ethanol at the end of the SM and NM fermentations are shown in Supplementary Table S3.

## Discussion

Yeasts are continually subjected to stressful environments due to non-optimum temperatures, lack of oxygen, acidity of medium or unbalanced nutritional composition, such as limited amounts of lipids, vitamins, nitrogen or mineral salts. Of all these stressors, many studies have revealed the importance of temperature on the growth of wine yeasts (Fleet, 2003; Beltran et al., 2006a; López-Malo et al., 2013; García-Ríos et al., 2014), and the influence of this environmental factor on determining the natural distribution of yeast strains and species during wine fermentation (Salvadó et al., 2011b; García-Ríos et al., 2014). Salvadó et al. (2011a) analyzed the thermotolerance of different *Saccharomyces* and non-*Saccharomyces* species using their growth kinetics parameters as measurable indicators. Their study was one of the first to show the cryotolerance of *S. kudriavzevii* and *S. uvarum* because these species displayed the lowest optimum temperature in the *Saccharomyces* genus. Most of the studied non-*Saccharomyces* also showed a lower optimum temperature than *S. cerevisiae*, except for *K. marxianus*. Temperature also seems to play the most important role in the imposition of *S. cerevisiae* versus non-*Saccharomyces* and non-*cerevisiae* species during wine fermentation (Salvadó et al., 2011b; Alonso del Real et al., 2017). Our results also highlight growth temperature as a key factor for separating *S. cerevisiae* from other tested species. More specifically, the phenotypic clustering showed that the wine *S. cerevisiae* strains grouped in a subcluster, which revealed very homogenous features in thermotolerance terms. Recent insights into the phylogeny of this species have revealed how human activity, and the anthropogenic niches in which have been isolated, have shaped the genomes and phenotypes of *S. cerevisiae* (Fay and Benavides, 2005; Legras et al., 2018; Peter et al., 2018).

Our competition experiment proved the imposition of two *S. uvarum* strains *versus S. cerevisiae* strains at low temperature, according to the obtained growth rate data. Nowadays, the use of strains belonging to other species of the genus *Saccharomyces* or non-*Saccharomyces*, either co-inoculated or sequentially inoculated with a *S. cerevisiae* strain, is becoming a popular practice in the wine industry. Hence the study of the parameters that determine the competitiveness of each strain and their permanence during wine fermentation is a most interesting topic for wine industry. According to our data, growth behavior under settled conditions could be considered a suitable predictor for the imposition of one strain on another in competition.

One smart alternative to this co-inoculation of strains is the formation of interspecific hybrids that combine the genome of the parental of interest. This is much easier for species of the genus *Saccharomyces*. In fact many natural interspecific *Saccharomyces* hybrids have been isolated from wine-related habitats (González et al., 2007; Lopandic et al., 2007; Lopes et al., 2010; Pérez-Través et al., 2015; Peris et al., 2016). Notwithstanding the above, the presence of genomic regions belonging to non-*Saccharomyces* species in the genome of *S. cerevisiae* wine strains has been reported (Novo et al., 2009; Galeote et al., 2010; Marsit et al., 2015). Unstable interspecific hybridization seems the most probable mechanism to explain these horizontal gene transfer events in yeasts (Marinoni et al., 1999; Guillamón and Barrio, 2017). In this work, we used a large collection of yeast strains of different species and origins to construct new hybrid strains. The best candidate for crossing with a low-cryotolerant wine *S. cerevisiae* strain was selected on the basis of thorough phenotyping and competition experiments with this strain collection. The parental with better fitness at low temperature belongs to the cryotolerant species *S. uvarum*. Many works have reported the successful hybridization of *S. cerevisiae* and *S. uvarum* and its use in enological conditions (Antunovics et al., 2005; Rainieri et al., 2006). Some works have also reported the biotechnological potential of this species to generate hybrids with *S. cerevisiae*, mainly for its capacity to grow at low temperature, but also for other enological features, such as low ethanol production together with high glycerol synthesis (Kishimoto, 1994; Zambonelli et al., 1997; Solieri et al., 2005; Lopandic et al., 2016; Origone et al., 2018). Despite most works using spore-to-spore crosses to generate interspecific hybrids, we have applied the rare-mating methodology. This methodology is based on the infrequent event of mating-type switching, which occurs in natural yeast populations (Spencer and Spencer, 1996), and can overcome low fertility associated with industrial strains (Codón et al., 1995). The hybrids generated by rare-mating often contain the complete DNA of both parents (Gunge and Nakatomi, 1972; Krogerus et al., 2016). After hybrid generation, the stabilization of new hybrid strains is very important. We used sporulation of hybrids in order to accelerate the genome reduction process, as the stabilization method. Notwithstanding sporulation has been widely reported as a genome destabilizing factor (Pfliegler et al., 2012; Karanyicz et al., 2017), by its use, we have obtained stable individuals more quickly than with other methods, further studies should be done in order to elucidate the genetic mechanism. Our results evidenced that the stabilization of the spores derived from an allotetraploid hybrid between *Sc* and *Su* occurred as 3n, likely alloaneuploid. Normal meiosis of an allotetraploid genome produces allodiploid spores. The intermediate genome size of S1–S3 could be due to chromosomal missegregations during tetraploid meiosis which can generate spores with various unbalanced combinations of parental chromosomes (alloaneuploid) (Kumaran et al., 2013; Mulla et al., 2014; Santaguida and Amon, 2015; Boynton et al., 2018). This fact is probably due to the lowest heterozygosity present in *Su* comparing with *Sc*, which seems to become the cells more prone to lose *Su* chromosomes (Almeida et al., 2014).

The importance of testing different individuals to generate sufficient genetic and phenotypic diversity is also noteworthy. This is because during the sporulation process, and as a result of meiosis segregation, several recombination events happen. Therefore, different combinations of alleles have been generated within the segregant population, which may possess distinct enological characteristics. This fact can be seen with spore S2, whose fermentative capacity is generally lower compared to S1 and S3. Nevertheless, the three hybrid segregants presented the most important characteristics of each parent, cryotolerance from *S. uvarum* in initial stages (short lag phases) and good fermentative capacity of the parent *S. cerevisiae*, especially in final stages when ethanol levels were higher. Even throughout the low-temperature process, hybrids performed better than parentals. This result is known as heterosis or hybrid vigor, which reveals that the arrangement of the genomes of both strains produce some combinations that improve fitness to make it superior to parentals (García-Ríos et al., 2017). Moreover, the biggest differences between segregants and parentals in fermentation length terms were observed in SM. SM was richer in yeast-assimilable nitrogen than NM. This discrepancy in nitrogen content may explain that segregants did not perform as well as in SM. It is well-known that nitrogen needs to increase at low temperature because plasma membrane rigidity results in less active membrane-associated permeases, and also in major membrane transport reduction (Beltran et al., 2006b; Pizarro et al., 2008; López-Malo et al., 2014). In any case, before being transferred to industry, the new strains should be tested in nutritional requirement terms to discover the best conditions to perform fermentations at low temperature.

## Conclusion

In conclusion, fermenting at low temperature is an accepted strategy to increase the final aroma of wines. However, it is still challenging for the wine industry to obtain yeast strains with good fermentation performance at a non-optimum temperature. Long and energetically expensive processes sometimes preclude the use of this enological practice in winery. The availability of genetic improvement techniques to construct new strains can partially supply this need of industry. Our work demonstrates that hybridization is an effective approach to obtain yeast strains with better fermentation performance at low temperature.

## Author Contributions

EG-R conducted the experiments, analyzed the data, and wrote the manuscript. AG and RdlC performed the experiments. LP-T helped in the study design. JG and AQ conceived the study, participated in the study design, and wrote the manuscript. All the authors read and approved the final manuscript.

## Conflict of Interest Statement

The authors declare that the research was conducted in the absence of any commercial or financial relationships that could be construed as a potential conflict of interest.
