# Supplementary material for: Improving the Cryotolerance of Wine Yeast by Interspecific Hybridization in the Genus Saccharomyces
Source: Front Microbiol. 2019 Jan 8;9:3232. doi: 10.3389/fmicb.2018.03232 (PMC6331415; doi:10.3389/fmicb.2018.03232)
Supplement: Supplementary file 3 [file Table_3.DOCX]

|  |  | **Synthetic must** | | | | **Merseguera** | | | |
| --- | --- | --- | --- | --- | --- | --- | --- | --- | --- |
|  |  | **Glucose (g L^-1^)** | **Fructose (g L^-1^)** | **Glycerol (g L^-1^)** | **Ethanol (% V/V)** | **Glucose (g L^-1^)** | **Fructose (g L^-1^)** | **Glycerol (g L^-1^)** | **Ethanol (% V/V)** |
| **15°C** | **71B** | 0±0 | 2.440±0.874 | 5.843±0.067 | 11.880±0.045 | 0±0 | 0±0 | 5.535±0.087 | 10.926±0.164 |
|  | **NPCC1314** | 0.117±0.101 | 7.370±2.563 | 6.133±0.117 | 11.013±0.146 | 0±0 | 0±0 | 7.433±0.160 | 9.481±0.100 |
|  | **H1** | 0±0 | 1.283±0.629 | 5.317±0.131 | 11.837±0.038 | 0±0 | 0±0 | 5.758±0.095 | 10.74±0.137 |
|  | **H2** | 0±0 | 1.283±2.223 | 5.130±0.156 | 11.693±0.093 | 0±0 | 0±0 | 5.835±0.077 | 10.803±0.005 |
|  | **H3** | 0±0 | 0.733±0.702 | 4.990±0.095 | 11.653±0.190 | 0±0 | 0±0 | 5.671±0.144 | 10.726±0.192 |
| **28°C** | **71B** | 0±0 | 0±0 | 6.780±0.046 | 11.927±0.153 | 0±0 | 0±0 | 7.418±0.867 | 10.748±0.071 |
|  | **NPCC1314** | 0.413±0.182 | 8.850±1.651 | 6.940±0.145 | 11.107±0.279 | 0±0 | 0±0 | 6.988±1.176 | 9.903±0.588 |
|  | **H1** | 0±0 | 0.820±0.314 | 7.487±0.958 | 11.393±1.409 | 0±0 | 0±0 | 7.17±0.180 | 10.67±0.160 |
|  | **H2** | 0±0 | 0.460±0.399 | 7.100±0.211 | 11.773±0.586 | 0±0 | 0±0 | 7.266±0.456 | 10.143±0.669 |
|  | **H3** | 0±0 | 0.530±0.089 | 6.947±0.006 | 11.663±0.071 | 0±0 | 0±0 | 6.488±0.449 | 9.481±0.929 |

**Table S3.** Main metabolite concentrations at the end of fermentation.
